# Barriers to Publishing Scholarship: A Study on Neurology Physician Residents and Fellows

**DOI:** 10.7759/cureus.71877

**Published:** 2024-10-19

**Authors:** Keng Lam, Kary M Calderon, Dowin Boatright, Jung G Kim

**Affiliations:** 1 Neuro-Oncology, University of Texas MD Anderson Cancer Center, Houston, USA; 2 Health Systems Science, Kaiser Permanente, Pasadena, USA; 3 Emergency Medicine, New York University, New York, USA

**Keywords:** all neurology, gme scholarly activity, neurology medical education, post-grad medical education, publishing

## Abstract

Background

Scholarly activity by trainees is required for US-accredited graduate medical education (GME) programs. Several factors, including financial barriers to open access (OA) journals, may impact trainees’ successful completion of scholarly activity, but little is known to what extent, particularly for neurology trainees.

Method

The authors implemented a cross-sectional, web-based 17-item survey of US-accredited neurology residency and fellowship programs during the 2022-2023 academic year. Participant responses for producing scholarly activity during GME were analyzed by mixed methods and examined by trainee motivation and perceived barriers, available institutional research support, and OA awareness and compared against socio-demographics (i.e., disadvantaged status history, underrepresented in medicine (URiM) status, international medical school graduate (IMG)) and prior research experience.

Results

Seventy-two respondents from 63 neurology programs completed the survey. Participants represented all US census regions and mostly from academic health centers and in advanced years of training. Overall, 17 (23.6%) self-reported as URiM and 20 (27.8%) as an IMG. Sixty-two (86.1%) were familiar with OA. Prior publications were associated with OA awareness (X^2^ = 5.3, p<0.05), and 27 (37.5%) reported financial barriers to publishing. IMGs reported less motivation to publish based on a journal’s impact factor (odds ratio [OR] = 0.15, 95% confidence interval [CI], 0.03-0.65, p<0.01) but were nearly 5 times more likely to report financial barriers to OA publishing (OR = 4.62, 95% CI 1.31-16.80, p<0.01). Trainees successfully publishing while training reported prior research experience (OR = 7.27, 95% CI 1.71-42.64, p<0.05) and access to mentors (OR = 4.67, 95% CI 1.52-14.64, p<0.001). Dedicated time for scholarly activity and publishing were reported as significant barriers in open-ended responses.

Conclusions

One-third of the study participants reported financial barriers to publishing scholarship, with these barriers disproportionately affecting IMGs. Prior research experience and access to mentors were associated with a higher likelihood of publishing.

## Introduction

Since scholarly activity has become a graduation requirement for accredited graduate medical education (GME) programs in the United States, more neurology residency programs are modifying their training programs to increase the publication output for their trainees [1,2]. Mentors, curriculum, and protected time have been frequently cited as important concepts to support scholarly activities [3]. Additionally, trainees will encounter peer-reviewed journals that offer open access (OA), a concept that facilitates timely scholarship, but OA publishing can be associated with substantial article processing fees [4].

Reports contextualizing the support for neurology trainees’ publishing scholarly activities from the lens of OA and socioeconomics barriers remain limited [5]. The purpose of this study is to describe the sociodemographic and GME program support factors that impact GME trainees’ scholarly activity in US-accredited neurology programs. We sought to evaluate factors associated with promoting or preventing the publishing of their scholarly activity, trainees’ awareness of the concept of OA, and how article processing charges have impacted trainees.

## Materials and methods

We designed a prospective mixed-methods study using a cross-sectional, web-based survey for trainees in accredited adult neurology residency and fellowship programs in the United States during the 2022-2023 academic year. The survey’s constructs on trainees’ awareness and perceived barriers were derived from a prior study at Memorial Sloan Kettering Cancer Center [6]. The confidential, web-based survey was designed in REDcap (Version 14.1.4, Oakland, California) and included 17 categorical items to measure the constructs of awareness (yes/no/unsure) and perceived factors, e.g., those that promote or prevent publishing, open-ended responses (Appendices) [7]. Prior to implementing the survey, we conducted a pilot with a sample of 10 content experts, neurology fellows, and research administrators to determine validity and iterated the survey after triangulating feedback from the pilot.

To identify eligible neurology programs, we queried the American Medical Association Residency and Fellowship Database (FREIDA) and reached out directly to 150 neurology programs’ program coordinators and program directors to distribute the survey [8]. We also recruited participants through publicly available professional social networks, including LinkedIn and Twitter. The survey period was open from December 30, 2022, until February 15, 2023. Study participants provided a unique survey code upon enrolling in the study. We offered a $100 gift card raffle as an incentive for respondents to complete the survey. The Kaiser Permanente Bernard J. Tyson School of Medicine Institutional Review Board approved the study (#1846156-1) as exempt research with minimal risk.

To characterize the sample, we analyzed respondents’ number of postgraduate years to date, whether the program was in an academic health center, the respondent’s program’s location based on the US Census regions, and the respondent’s socio-demographics i.e., history of disadvantaged status as defined by the Association of American Medical Colleges underrepresented in medicine (URiM) status, international medical school graduate (IMG), and awareness of OA publishing [9]. To integrate characteristic data about the respondents’ neurology residency programs, we linked survey respondents’ responses to the FREIDA. We then analyzed respondents’ success in publishing during their GME, motivation factors and perceived barriers, available institutional research support, and OA awareness for producing scholarship during GME for associations with socio-demographic factors. Associations of factors were analyzed by chi-squared (Χ^2^) tests and odds ratios (OR). Quantitative data were analyzed using STATA 17.0 (Stata Corp, College Station, Texas). Respondent comments were analyzed for themes to supplement our quantitative data findings.

We report our findings in accordance with Strengthening the Reporting of Observational Studies in Epidemiology (STROBE) guidance [10].

## Results

As shown in Table 1, 72 neurology trainees from 63 programs completed the survey. Participants represented all US census regions, were mostly from academic health centers 54 (75%), and with an average of 3.5 years of GME training (+/- 1.22 postgraduate years). Overall, 17 (23.6%) participants self-identified as URiM and 20 (27.8%) as an IMG. Most were familiar with OA publishing (n = 62; 86.1%).

**Table 1:** Demographics of Respondents (N = 72) a. Differences found at p<0.05 by the X^2^ test b. We used the American Association of Medical College’s definition of underrepresentation in medicine as “racial and ethnic populations that are underrepresented in the medical profession relative to their numbers in the general population.” GME: Graduate medical education

Respondent characteristics, n (%)	Published during GME training, n = 37	Has not published during GME training, n = 35
# of Postgraduate Years^a^,(mean, SD)	3.9 (0.19)	3.1 (0.2)
Attending a GME program or fellowship in the academic health center	28 (38.9)	26 (36.1)
No	9 (12.5)	9 (12.5)
Census region		
Midwest	9 (12.5)	7 (9.7)
Northeast	14 (19.4)	6 (8.3)
South	10 (13.9)	14 (19.4)
West	4 (5.6)	8 (11.1)
Underrepresented in medicine^b^	7 (9.7)	10 (13.9)
No	30 (41.7)	25 (34.7)
Self-reported history of socioeconomic and disadvantaged status	7 (9.7)	9 (12.5)
No	29 (40.3)	25 (34.7)
International medical graduate	10 (13.9)	10 (13.9)
No	27 (37.5)	25 (34.7)
Aware of open access publishing^a^	35 (48.6)	27 (37.5)
No	2 (2.8)	8 (11.1)

Prior success with publishing was associated with OA awareness (X^2^ = 5.3, p<0.05), and 27 (37.5%) reported financial barriers to publishing their scholarly work. As shown in Table 2, both IMGs (OR = 0.15, 95% confidence interval [CI] 0.03-0.65, p<0.01) and URiM (OR = 0.2, 95% CI 0.50-0.88, p<0.01) reported less motivation to publish based on a journal’s impact factor. IMGs were nearly 5 times more likely to report financial barriers to publishing in OA journals (OR = 4.62, 95% CI 1.31-16.80, p<0.01). Trainees who successfully published while in training reported prior research experience (OR = 7.27, 95% CI, 1.71-42.64, p<0.05) and access to mentors (OR = 4.67, 95% CI 1.52-14.64, p<0.001).

**Table 2:** Comparison of Motivational Factors and Barriers for Publishing During GME Training a. Differences found at p<0.05 by the odds ratio test. b. Respondents all responded yes to this item. GME: Graduate medical education

	Published during GME training, OR (95% CI)	History of disadvantaged status, OR (95% CI)	Under-represented in medicine, OR (95% CI)	International medical graduate, OR (95% CI)
Motivation to scholarly work				
Program requirement	0.41 (0.14-1.16)	0.56 (0.15-1.98)	0.68 (0.19-2.31)	0.35 (0.09-1.2)
Prior research training	7.27^a^ (1.71-42.64)	0.63 (0.10-2.79)	1.34 (0.31-5.12)	0.45 (0.07-1.94)
Residency or fellowship curriculum or resources	0.81 (0.27-2.45)	1.37 (0.35-4.99)	0.86 (0.20-3.14)	0.49 (0.10-1.87)
Research mentors available	4.67^a^ (1.52-14.64)	0.65 (0.18-2.63)	0.40 (0.11-1.39)	0.49 (0.15-1.64)
Prospects for career promotion	2.21 (0.77-6.42)	1.25 (0.35-4.88)	0.75 (0.22-2.62)	0.49 (0.15-1.63)
Factor for where to submit scholarly work				
Journal’s impact factor	1.42 (0.45-4.60)	0.56 (0.15-2.23)	0.90 (0.24-3.83)	0.53 (0.15-1.97)
Journal audience in my specialty or research area	1.53 (0.40-6.05)	0.36 (0.08-1.71)	0.21^a^ (0.50-0.88)	0.15^a^ (0.03-0.65)
Potential costs needed to publish	0.85 (0.28-2.52)	0.56 (0.16-2.10)	0.89 (0.25-3.43)	2.42 (0.63-11.33)
Public accessibility of journal	0.41 (0.13-1.30)	1.04 (0.24-3.91)	1.33 (0.34-4.75)	0.53 (0.11-2.06)
Would publish if open access option was available	^b^	^b^	^b^	^b^
Experienced financial barriers when publishing scholarly activity	1.82 (0.63-5.25)	2.14 (0.60-7.81)	2.06 (0.55-7.56)	4.62^a^ (1.31-16.80)

The participants also provided open-ended responses regarding barriers and support during their GME training period. Time needed for research and publishing were reported as significant barriers. One respondent reported, “Residency time is busy and sometimes not having dedicated time to do research counts. Most of my time is patient care and academic responsibilities as well as taking care of my family.” Another respondent added, “The research I published while in residency so far was based on research I completed while in medical school... Since starting residency, it has been difficult to complete a research project for publication so far due to the demands of clinical care in residency.”

We found other themes related to journal submission fatigue and time demand, including rejection from journal decisions. One respondent stated, “Submission fatigue-if one journal rejects it can be difficult to want to reapply somewhere else with all the reformatting requirements.”

Financial barriers reported included high publication costs and limited funding provided by GME programs. One trainee stated, “[I was] considering submitting to a journal but they charged too much for the program to cover.” Another added, “Publication costs are not trivial and help is not offered by [my] parent institute.” Those with smaller or fewer financial barriers may have their publication costs offset, with one trainee stating, “Peer review published articles are reimbursed up to $300 per published article, per each resident, reimbursed at a maximum of one time during the length of the resident's program specialty training.” Fees covered by trainees’ mentors were also reported as helping to overcome financial barriers to OA publishing.

## Discussion

This exploratory study contextualizes the barriers to the required ACGME scholarly activity in adult neurology trainees. While previous surveys on GME trainees have identified barriers to conducting research studies, those studies did not explore factors related to publishing with respect to completing a training program’s scholarly activity requirement [11,12]. Our study provides an early and important insight into the current state of required scholarly activity from a neurology trainee’s perspective.

We found that one-third of participants reported financial barriers to publishing. These findings have not been previously reported to our knowledge. Article processing charges may create a financial challenge for trainees who may not have access to funding. For example, neuroscience peer-reviewed journals that are exclusively OA require over $2,100 USD to publish [13]. For journals in emergency medicine and critical care, the average article processing charge exceeds $2,200 USD [14]. While many training programs have professional development funding (e.g. for trainees to travel to conferences to present their research), it is unclear to what extent GME programs reimburse for article processing charges on behalf of trainees.

Our study’s disproportionate sample of URiM trainees (23.9% compared to 12.5% nationally in GME) may suggest an interest among this group to share their professional development barriers experienced in GME, and offering potential future questions on how URiM trainees navigate their professional development [15]. Our study also shows IMG trainees were less concerned with a journal’s impact factor; we hypothesize that there are social pressures for pursuing any reputation-building activities to advance their professional careers, which would require further study [16]. Attempts at publishing for IMG trainees add to other existing challenges while training in US-based GME programs, which may negatively affect trainee well-being [17]. Our findings also highlight the importance of amplifying the voices of URiM and IMG trainees through future research.

Our study also found that access to research faculty mentors is associated with a higher likelihood of publishing scholarly activity during GME training. This finding is consistent with a previous report identifying strong mentorship as an important contributing factor for research productivity [18]. Ensuring the availability of research mentorship in GME programs and fellowships is needed given the requirement that all GME programs must provide ample scholarly activity opportunities. Future studies on best practices in meeting the scholarly activity requirement in GME should consider the accessibility of resources (e.g., time and mentors) to optimize success for residents and programs. These studies could lead to improved national standards for addressing barriers to scholarly activity requirements. Future studies could investigate whether programs with experienced research faculty, dedicated professional development funding to support trainees to present and publish their scholarly work, and elective time to promote scholarly activity during GME [19].

Our study findings are limited by potential self-selection bias and the small sample size of adult neurology trainees who responded to the surveys after extensive virtual outreach. However, to our knowledge, this is the first independent study to explore the issue of publication barriers for GME trainees. Our study also provided free responses to highlight the issues faced by the trainees on publishing.

## Conclusions

One-third of the study participants reported financial barriers to publishing scholarship, with these barriers disproportionately affecting IMGs. Prior research experience and access to mentors were associated with a higher likelihood of publishing. Supporting neurology residents and fellows in scholarly activity during GME will require training programs, their sponsoring institutions, and national bodies to operationalize resources to realistically meet the scholarly activity requirement and help promote the GME workforce as scholars. Future work should consider collaborating with larger national bodies, such as the ACGME and American Association of Colleges of Osteopathic Medicine, to explore similar research questions for trainees across all medical specialties.

## Appendices

Survey

**Table 3:** Survey Questions

Barriers to Publishing: A Cross-Sectional Study for Residents and Fellows
Are you a currently active neurology resident/fellow for the 2022-23 academic year?
☐ Yes
☐ No
If No, thank you for your participation.
What post-graduate year are you in?
☐ PGY1
☐ PGY2
☐ PGY3
☐ PGY4+
☐ Fellow
What is the complete name of your GME program? ______________________
Location of your GME program? Zip code:___________
What medical/osteopathic school did you attend? ___________
Have you ever published in a peer-reviewed journal?
☐ Yes
☐ No
☐ Unsure
Have you published in a peer-reviewed journal at any point during your residency and/or fellowship training?
☐ Yes
☐ No
☐ Unsure
Which of the following is a motivation for you to complete your required scholarly work during your residency and/or fellowship training? (Select all that apply)
☐ Program requirement
☐ Prior research training/formal degree
☐ Research curriculum/resources at current program
☐ Research mentors available
☐ Increase prospects for career promotion/further GME training
☐ Other: ___________
Does your program or sponsoring institution offer support to help publish your work in a peer-reviewed journal.
☐ Yes: please describe___________________
☐ No: please describe___________________
☐ Unsure: please describe___________________
Which of the following are factors you would consider in deciding where to submit your scholarly work? (Select all that apply)
☐ Impact Factor
☐ Journal audience is in my specialty/research topic area
☐ Potential costs related to publishing your research
☐ Accessibility to the public
☐ Other: ___________
Have you heard of the option of “open access” when it comes to peer-reviewed publications?
☐ Yes
☐ No
☐ Unsure
Would you publish your scholarship in an “open access” issue if that option was made available to you?
☐ Yes
☐ No
☐ Unsure
Have you faced financial barriers (e.g. article processing charges for authors) in an attempt to publish your research?
☐ Yes: please describe___________________
☐ No: please describe___________________
☐ Unsure: please describe___________________
What obstacles have you faced in the process of finding a peer-reviewed journal to publish? Please describe: _______________________
Would you consider yourself Underrepresented in Medicine? *
*The American Association of Medical Colleges defines "Underrepresented in medicine means those racial and ethnic populations that are underrepresented in the medical profession relative to their numbers in the general population."
☐ Yes
☐ No
☐ Unsure
Would you consider yourself as an individual with a history of socioeconomic and disadvantaged status at any point while pursuing a career in medicine?*
☐ Yes
☐ No
☐ Unsure
(optional) Any other experiences with your pursuits of scholarly activity while in GME that you’d like to share. Please describe:_______________________
